# Phylogenetic Analysis of *Elaeagnus* L. in China: A Basis for Genetic Improvement of a Berry Crop

**DOI:** 10.3389/fpls.2022.899079

**Published:** 2022-06-09

**Authors:** Chunsong Cheng, Siqing Fan, Canjian Wang, Linlin Ye, Zupeng Wang, Hongwen Huang

**Affiliations:** ^1^Lushan Botanical Garden, Chinese Academic of Sciences, Jiujiang, China; ^2^Jiangxi University of Chinese Medicine, Nanchang, China; ^3^School of Applied Science and Technology, Hainan University, Haikou, China

**Keywords:** underutilized fruit, fruit crop, morphological clustering, molecular phylogeny, phylogenetic tree

## Abstract

*Elaeagnus* L. is found in wild or grown as ornamental plants and is increasingly regarded as underutilized berry shrubs by breeders. This genus has cosmopolitan distribution with various species widely distributed in China, Europe, the United States, and Canada. Interspecific hybrids, which have been reported several times, have attracted intense interest from plant breeders attempting to develop a fruit crop of *Elaeagnus*. Orthogonal projections to latent structures discriminant analysis (OPLS-DA) is a powerful statistical modeling tool that provides insights into separations between experimental groups. In this study, the molecular phylogeny of *Elaeagnus* species was first discussed using the ITS and matK sequences for guiding the construction of a genetic basis pool. A morphological OPLS-DA clustering model based on the genetic divergence was also constructed for the first time, which effectively realized the morphological grouping of Chinese *Elaeagnus* species. The results showed that a total of 10 wild species widely distributed in China have the potential to develop fruit crops. Particularly, *Elaeagnus conferta* has the potential to provide a founder species with a large fruit size, while *Elaeagnus Gonyanthes* has the potential to provide important genetic resources with long pedicel. *Elaeagnus lanceolata* and *Elaeagnus delavayi* could be used to domesticate hybrids without spines, and the other five climbing shrubs could be used to develop high-yield crown-type commercial cultivars for automated field management. The top five contributing morphological traits affecting the current clustering model were V9 (flower color), V1 (flowering), V5 (evergreen or deciduous), V3 (leaf size), and V2 (fruiting). Furthermore, the grouping analysis indicated that the V9 was the most important factor affecting morphological clustering. Thereafter, the temporally calibrated phylogeny inferred from the matK sequence was used to reconstruct the origin and evolution of the genus *Elaeagnus*, and the results inferred an interesting geographic distribution pattern and potential cross-species interactions of *Elaeagnus* species at low latitudes in China. Our study also highlighted dispersal pattern investigation and genetic background analysis to improve future practices and policies related to species introduction of genetic basis pool.

## Introduction

*Elaeagnus* L., a genus of the Elaeagnaceae, with about 100 recognized wild species, is cultivated as an ornamental or a fruit crop for its dense shrub-like structure, fragrant flowers, and lycopene-rich ripe fruits (Sun and Lin, 2010; Alexandrov and Karlov, 2021). The genus *Elaeagnus* is native to temperate and subtropical regions of Asia, Australia, southern Europe, and North America (Ye et al., 2012). In order to better understand the worldwide use of this genus, a detailed table has been performed and gives a clear representation of medicinal and edible applications in China, South Korea, India, Australia, and other countries. As shown in Table 1, there are 15 species, including an endangered woody oil species, *Elaeagnus mollis*, in this genus that has been widely reported to have valuable edible or medicinal properties. As an endemic berry fruit, only seven juicy berries are considered underutilized fruits, including *Elaeagnus conferta* (Dandge et al., 2011; Jaafar et al., 2018), *Elaeagnus kologa* (Sasikumar et al., 2012; Patel, 2015), *Elaeagnus latifolia* (Dasila and Singh, 2021), *Elaeagnus multiflora* (Lee et al., 2011), *Elaeagnus pyriformis* (Banerjee et al., 2022), *Elaeagnus trifloral* (Ramadevi and Narayana, 1990), and *Elaeagnus umbellata* (Khattak, 2012). The red berries are known as oleaster, silverberry, autumn olive, thorn olive, Russian olive, Persian olive, and wild olive (Patel, 2015). Currently, these species are usually planted in gardens or around courtyards as ornamental shrubs in most countries and regions, and the sweet, rare fruits are nowadays only regarded as a kind of accessory product. Surprisingly, few varieties have been developed for pomology purposes although this genus has the potential to develop into a great fruit crop for the benefit of the world.

**TABLE 1:** The widely reported species in *Elaeagnus* L. with valuable edible or medicinal properties.

No	Name	Native	Wild or Not	Use	References
1.	*E. angustifolia* Russian olive	Eurasian tree natives to Turkey and invaded zones along watercourses all over the world.	W	Antioxidant capacity	Ahmadiani et al., 2000; Klich, 2000
2.	*E. commutata* Silverberry	North America	W	Ornamental shrub rangeland weed	Corns and Schraa, 1962; Chwil and Weryszko-Chmielewska, 2011
3.	*E. conferta*	China	W, C	Underutilized fruit	Liu et al., 2019
4.	*E. glabra*	Korea	W	Medicinal plant	Li et al., 2009; Chen C.-Y. et al., 2019; Sohn et al., 2019
5.	*E. kologa*	The western ghats in India	W	Endemic fruit	Paulsamy et al., 2010; Sasikumar et al., 2012
6.	*E. latifolia* Soh-Shang	Northeast India Thailand and also in Vietnam	W	Wild edible fruit underutilized fruit	Patel et al., 2008; Panja et al., 2014
7.	*E. macrophylla*	East Asian coastal areas and islands	W	Coastal greening	Wang et al., 2020
8.	*E. mollis*	the Loess Plateau of China	W, C	Endangered species woody oil plant	Liang et al., 2015; Gao et al., 2021
9.	*E. multiflora* cherry silverberry	China and Japan	W	Underutilized fruit decorative fruit used in traditional Chinese medicine	Ismail et al., 2015; Lizardo et al., 2020
10.	*E. oldhamii*	Mainly used in Taiwan	W	Traditional herbal medicine	Liao et al., 2013, 2014
11.	*E. pungens* thorny olive	Native to China and Japan introduced to the eastern and southeastern United States	W, C introduced into the United States in 1830	Ornamental plant traditional Chinese medicine	Hu et al., 2016; Qi et al., 2021
12.	*E. pyriformis*	Northeast India	W	Underutilized fruit underutilized juicy fruit-bearing	Khomdram et al., 2014; Kar et al., 2016
13.	*E. triflora* bennaken	Extends to northeastern Australia from Asia south	W, C	It is often grown in gardens as a fruit plant, also described to children sick by dysentery	Abdalla, 2019
14.	*E. umbellate* autumn olive	Native to Asia but introduced to North America and spread from cultivation in the mid- and the eastern United States	W, C naturalized	Edible fruit	Fordham et al., 2001; Naumann et al., 2010
15.	*E. rhamnoides* sea buckthorn	Native to Europe and Asia	W, C	Oil plant;Spiced juice	Selvamuthukumaran and Khanum, 2014; Olas, 2018

*W, wild; C, cultivated.*

Nevertheless, it is interesting that the selection and breeding based on the excellent genetic material of *E. conferta* have been developed for nearly 20 years in mainland China (Gill and Gupta, 2018; Liu et al., 2019). *E. conferta* has an absolute advantage in fruit size, but distribution is limited in China’s low latitude subtropical regions, such as Yunnan and Guangxi Provinces. Except for the populations of *E. conferta*, nearly 55 species of this genus are also widely distributed in China, spread from the Hexi Corridor to the Yangtze River Basin to mountain areas of southern China (Sun and Lin, 2010). Self-incompatibility is a common feature of *Elaeagnus* plants, which offers the possibility of creating new cultivars through interspecific hybridization. In fact, the World Flora Online (WFO) and the Plant List (TPL) and recent reference (Abdalla, 2019) have recorded 11 hybrids with three accepted new hybrid cultivars of the genus *Elaeagnus*, namely *E.* × *reflexa* E. Morren & Decne., *E.* × *submacrophylla* Servett., and *E.* × *maritima* Koidz. A popular horticultural hybrid (Li Y. L. et al., 2015), gold-marginatus hybrid *E.* × *ebbingei* Boom., “Gilt Edge” (syn. *E.* × *submacrophylla*) could provide an inspiring example for creating new hybrid cultivars. It’s reported that the gold-marginatus hybrid *E.* × *ebbingei* (Chen and Wu, 2019) was first created by interspecific hybridization in Netherlands in 1929, and the two parents selected were *Elaeagnus pungens* and *E. macrophylla*, which are both widely distributed across multiple latitudes in the southeast coast of China (Alexandrov and Karlov, 2021). Therefore, the creation of new fruit crops of *Elaeagnus* is inseparable from the ingenious collection and selection of wild resources under a superb scientific design.

Hongwen et al. (2021) pointed out that conventional breeding methods, especially natural mutant selection, hybrid breeding, and systematic breeding are still the preferred ways for the initial domestication of new fruit crops. Among them, recurrent selection (Payne et al., 1986; Cobb et al., 2019) is an important breeding method to improve the population of crops, especially the population of cross-pollinated species. Since large-scale geographically adaptive distribution is an essential condition for the selection of hybrid fruit crops with desirable characteristics, an effective basis pool filled with more genetic resources needs to be established to select and breed the next generational fruit-type *Elaeagnus* crops (Cornille et al., 2015). Therefore, how to rapidly and efficiently construct primitive populations by using the local and cross-regional relatives in the same genus has become an urgent consideration for selection design. It is the general consensus now that the progeny of interspecific hybridization is difficult to be obtained in a wide cross because of their strong cross-incompatibility (Lexer et al., 2003; Maune et al., 2018). In order to achieve fertile recombinant progenies, the primary condition of parental selection is to overcome this strong barrier. Experimental crosses, as a tool for studying breeding in the wild, provide a way to overcome natural barriers, as the traits most important for adaptation and speciation may be fixed in wild populations (Lexer et al., 2003). However, the selection of wild resources of perennial shrubs requires a lot of time for resource collection and selective mating experiments. Which traits contribute most strongly to assortative mating specifically and to reproductive isolation more generally?

In most cases, molecular phylogenetic analysis and morphological clustering are reliable methods for providing genetic relationships and predicting reproduction compatibility among plant species (Baral and Bosland, 2004; Kubota et al., 2012). Currently, several phylogenetic studies have provided credible insights for understanding the evolutionary relationships of *Elaeagnus*, and the consensus trees supported the monophyletic origin of the genus *Elaeagnus* and *Hippophae* (Bartish et al., 2002; Kim et al., 2020; Zhao et al., 2020). Son et al. (2014) provided the molecular phylogenetic relationships of nine common Asian *Elaeagnus* species based on nrDNA ITS sequences. Their study reported that different clustering methods can be used for obtaining similar divergent results, except for the ambiguity in the divergence time of the outer taxa in the genera *Shepherdia* and *Hippophae.* Besides, the strict consensus trees based on cpDNA and morphological characters of the Elaeagnaceae showed that the genera *Elaeagnus* and *Hippophae*are are similar in chloroplast DNA sequence (Zhao et al., 2020). Phylogenetic trees constructed by the maximum likelihood (ML) method based on complete chloroplast or plastid genomes also showed that the genus *Elaeagnus* has a close relationship with the genus *Hippophae* (Choi et al., 2015; Liu et al., 2019; Cheng et al., 2020; Kim et al., 2020). At present, more phylogenetic and molecular clock analysis of Chinese *Elaeagnus* using DNA marker regions in the chloroplast genome, such as matK, rbcL, trnH-psbA, and trnL-F, has not been reported yet. A more rigorous and phylogenetic analysis is very important for further resource evaluation. Recently, many studies (Yu et al., 2011; Cabelin and Alejandro, 2016) have reported that the matK (cpDNA) sequence has significant stability in phylogenetic analysis, and the constructed genetic relationship is closer to the real plant taxonomy. In this study, DNA markers in the nuclear genome, such as ITS, and that in the chloroplast genome, such as matK, should both be used to perform comparative analysis to obtain biparental and maternal characteristics of *Elaeagnus* species, and provide the theoretical basis for further crossbreeding. Moreover, because the phylogenetic signals provided by molecular phylogenetic results are prone to contradictory taxonomic results (Eernisse and Kluge, 1993), morphological clustering analysis is also important to infer the genetic relationship among the *Elaeagnus* species. Additionally, morphological clustering under a grouping condition of strict molecular phylogenetic clades may provide a new means to infer the interspecies cross-compatibility. Principal component analysis (PCA) and orthogonal projections to latent structures discriminant analysis (OPLS-DA) are powerful statistical modeling tools that provide insights into separations between experimental groups. OPLS-DA has spawned a general pattern in many fields. When PCA fails to identify significant separation between experimental groups, analysts may move to construct OPLS-DA models. OPLS-DA provides an avenue for predicting group membership based on a set of high-dimensional measurements and holds many advantages over PCA. So, we used the clades directly supported by the phylogenetic tree as the experimental grouping for morphological data grouping, which provided a potential training dataset for building OPLS-DA models.

In this study, phylogenetic analysis was conducted for the genetic grouping of wild species in the genus *Elaeagnus* by using the public sequencing data of DNA marker regions in the chloroplast genome. Then, an OPLS-DA model based on 12 morphological traits was established for all Chinese *Elaeagnus* species. In addition, the geographic dispersion of multilateral co-evolution was analyzed for the first time and was expected to be used to guide the selection of founder parents and the construction of basis pools for the selection and breeding of new *Elaeagnus* fruit crops.

## Materials and Methods

### Sampling, Sequence Acquisition, and Processing

All DNA sequences including ITS and matK of *Elaeagnus* L. were downloaded from GenBank in NCBI. Sequence alignment was performed by using the BioEditor, and all sequences were aligned using Clustal W. Sequence deletions or errors exceeding 5% of the total bases were considered low-quality sequences and were discarded. Gaps were treated as missing data. The same species retained all haplotypes for further phylogenetic analysis. The head and tail of the aligned sequences were cut off. In order to ensure DNA sequence data were correctly linked taxonomically to the assigned species, we only retained haplotypes with more than 99% sequence similarity of the same species for further analysis. Thereafter, as shown in Supplementary Table 1 and Table 2, 56 ITS sequences of at least 541 bp and 53 matK sequences of at least 689 bp were selected for further processing, the full range of ITS and matK were shown in the Supplementary Table 2. Among them, the sequence of outgroup was given by a member of the Rhamnaceae (e.g., *Ziziphus jujuba* Mill.), 50 ITS sequences of *Elaeagnus* species were used for phylogenetic analysis together with five ITS sequences of *Hippophanae* species, and 53 matK sequences of *Elaeagnus* species were used for phylogenetic analysis together with eight matK sequences of *Hippophanae* species. Additionally, three hybrids and one environmental variety were the main focus in order to find pieces of evidence of cross-compatibility and interspecific hybridization. Finally, the software Geneious_11 was used for constructing the molecular matrix (Kearse et al., 2012).

**TABLE 2:** Ten resources of Chinese wild *Elaeagnus* L. species with the potential to develop into fruit crops.

No.	Latin name	Dominant traits	Fut. Size	Plant type	Fl.
1.	*E. glabra*	Widely distribution	14–19 mm	Evergreen, climbing, shrubs	Sep-Nov
2.	*E. gonyanthes*	Long pedicel	15–22 mm	Evergreen, climbing, shrubs	Oct-Nov
3.	*E. henryi*	—	5–8 mm	Evergreen, climbing, shrubs	Oct-Nov
4.	*E. macrophylla*	Less flowers	15–20 mm	Evergreen, slightly climbing, shrubs	Sep-Nov
5.	*E. conferta*	Desirable fruit size	20–40 mm	Evergreen, straggling, shrubs	Oct-Nov
6.	*E. lanceolata*	Spines absent	12–15 mm	Evergreen, erect or divaricate, shrubs	Aug-Oct
7.	*E. delavayi*	No spines	6–12 mm	Evergreen, erect, shrubs	Sep-Dec
8.	*E. oldhami*	Special fruit shape	7–8 mm	Evergreen, erect, shrubs or small trees	Nov-Dec
9.	*E. pungens*	Widely distribution	12–15 mm	Evergreen, erect, shrubs	Sep-Dec
10.	*E. bockii*	Desirable shrubs type	10–12 mm	Evergreen, erect, shrubs	Oct-Nov

### Quantitative Phenotype

Referring to “Flora of China (FOC)” (Flora of China Editorial Committee, 2018), “Higher plants in China,” and “Image of higher plants in China,” all taxonomic descriptions were quantitatively summarized and analyzed. Operationally, we first extracted all morphological traits recorded in FOC, and after classification, quantification, and data standardization, we finally identified 12 traits related to life form, ecotype, pollination ecology, and reproductive ecology of *Elaeagnus* species. A total of 12 investigated traits were flowering, fruiting, the minimum leaf size, the maximum leaf size, evergreen or deciduous, plant height, spines or absent, climbing or not, flower color, the length of calyx tube, the style villous, and the flower number. The quantitative methods by chronological order or degree were used to obtain the quantitative data. In this section, 55 species were investigated by using high-resolution specimen pictures online. Specimens of the same species from at least six different regional sources were used to extract and quantify trait information. SPSS 22 (IBM, China) was used for data standardization (Leech et al., 2014).

### Sequence Divergence and Phylogenetic Analysis

All DNA sequences including ITS and matK were first aligned with MEGA-X (iGEM, France). Since many sequences have a small number of base deletions (often denoted as “NNNN…”). Then, the consensus sequences were assembled and visually checked for quality using SeqScape version 2.5 (Applied Biosystems, Foster, United States). Phylogenetic reconstruction was performed by using maximum parsimony (MP), ML, and Bayesian inference methods to resolve the interspecific and intergeneric phylogenetic relationships. For ITS and matK sequences, MEGA-X (iGEM, France) was used for conducting MP or ML analyses with 1,000 bootstrap replicates (Kumar et al., 2018), and the best model for constructing an ML tree by using MEGA-X (iGEM, France) is Hasegawa–Kishino–Yano + Gamma Distributed (G) model or Kimura 2-parametric (K_2_P) model, relying on the calculation results from Find Best DNA/Protein Models (Srivathsan and Meier, 2012). The time-calibrated phylogeny inferred from the matK sequences was using BEAST 2.2 (Bouckaert et al., 2019) with an uncorrelated rates model. BEAST 2.2 analysis was conducted under the guidance of the online tutorial. First, the Bayesian Evolutionary Analysis Utility (BEAUti) was used for setting the evolutionary model and options for the Markov chain Monte Carlo (MCMC) analysis. Because K_2_P models are not embedded within BEAST 2.2, the BEAUti interface was used in which a selected model (GTR + G) for the aligned dataset (nex or fasta file) was applied with a Yule speciation tree prior and an uncorrelated lognormal molecular clock model. Two runs of 100 million generations of MCMC chains were produced, sampling every 1,000 generations. Then, BEAST was running using the input file that contains the data, model, and settings. We used the following three constraints for fossil calibration (all with a normal prior distribution): Elaeagnus stem node 30 ± 6 Ma (Akgiin and Sözbilir, 2001), *Hippophanae*crown node 0.39–0.1 Ma (Ruixue, 2013), and *Elaeagnus* crown node 11.2 ± 4 Ma (Su et al., 2014). Following convergence, the resulting trees were combined using TreeAnnotator (BEAST Developers). The final phylogenetic tree was visualized using the FigTree (Softpedia, Romania). The phylogenetic tree was visualized by EVOLVIEW (EvolGenus Info., Online) (Subramanian et al., 2019).

### Orthogonal Projections to Latent Structures Discriminant Analysis Model Construction

According to phylogenetic grouping, normalized data of 12 morphological traits were used to construct the OPLS-DA model. PCA without group setting was used to compare and support the advantages of the OPLS-DA method. The receiver operating characteristic (ROC) curve was typically used to evaluate the effectiveness of the grouping boundary, and the area under the ROC curve (AUC) is regarded as a measure of the overall performance of a diagnostic test and is interpreted as the average value of sensitivity for all possible values of specificity (Obuchowski, 2003; Zhou et al., 2009). AUC is often presented along with its 95% confidence interval (CI). Therefore, if the lower bound of the 95% CI of AUC for a test is greater than 0.5, then the test is statistically significantly better. The variable importance for the projection (VIP) summarized the importance of the variables and was sorted to display larger VIPs to the left. The top 5 contributing quantitative traits affecting the current clustering model were further analyzed by the *F*-test and the *t*-test.

### Analysis of Geographic Dispersion

Refer to FOC and the specimen records in “Chinese Virtual Herbarium”^1^ and as shown in Supplementary Table 3, specimen collection sites were counted, and the frequency of occurrence of specimens in the same geographic area was used to determine the distribution center of each species. The main distribution centers of different species in different groups separated by the OPLS-DA model were analyzed using ArcGIS 10.8 (Esri, United States). Neural network analysis based on Gephi 0.9.2 (Gephi.org, Atlanta, GA, United States) was used to better visualize the geographic dispersion of *Elaeagnus* species in China. The nodes include Latin names of species, group names, and five geographical regions of China, such as East China, Central China, South China, Southwest, and North China.

### Statistical Analysis

Excel as a basic tool was used to record and edit the data and convert file formats. Graphpad Prism 7 software (GraphPad Software Inc, San Diego, CAï United States) was used to analyze the data. All numerical values are presented as mean ± *SD*. R and open-source analysis packages were used for difference analysis, such as the *t*-test, *f*-test, mean, and the Kruskal–Wallis test. Statistically significant differences were determined by pair using a *t*-test, with *p*-values < 0.05 or 0.01.

## Results

### Molecular Phylogenetic Analysis for Exploring Reliable Divide Criterions

**FIGURE 1:**
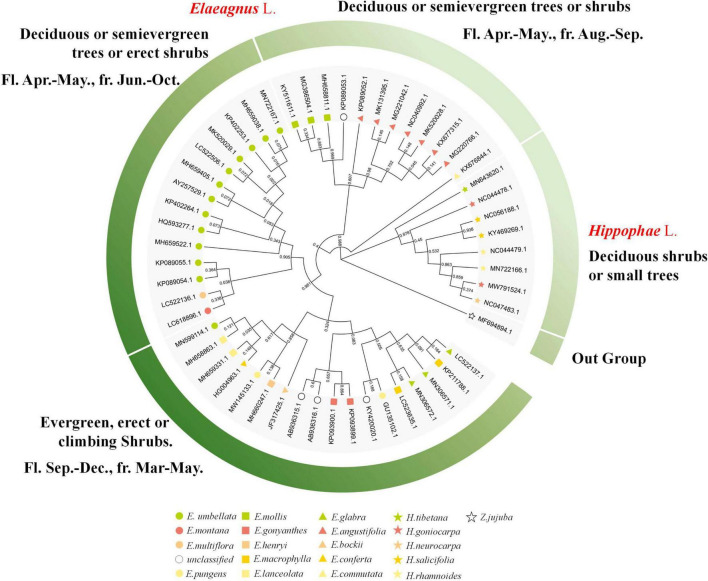
Phylogenetic tree combined with the main plant taxonomic features constructed with the matK sequences for *Elaeagnus* plants (Bootstrap values are expressed as decimals).

The public sequences of *Elaeagnus* and *Hippophae* were collected for molecular phylogenetic analysis so as to facilitate the comparative analysis with traditional plant taxonomy. Two characteristic DNA marker sequences, including matK and ITS sequences, were obtained as the basic data to construct phylogenetic trees. In this study, the matK sequences of 21 species and ITS sequences of 25 species were used to construct the matrixes and perform molecular phylogenetic analysis. Among them, 109 parsimony characters were found in the ITS matrix, and 45 parsimony characters were found in the matK matrix. With *Z. jujuba* as the outgroup, the MP based on the ITS and the ML based on the ITS and matK were used for constructing the strict consensus trees (Supplementary Figure 1). Both trees revealed that *Hippophae* L. was divided into an independent clade with a bootstrap value of more than 95%. Therefore, both trees were invaluable for further analysis of genetic classification. Since the ITS is biparental and the matK is maternal, so the molecular phylogenetic results based on ITS sequences could better reflect the genetic relationship between hybrids and their parents. ITS tree analysis by MP or ML methods showed that three hybrids and one environmental variety were divided into one clade together with *E. pungens, E. glabra, E. macrophylla, E. conferta, E. angustifolia, E. multiflora*, and *E. umbellata*, which were mentioned in Table 1 and considered to be underutilized fruits or widely used cash crops. Therefore, the concerned seven species have the potential to become the founder species or precious wild resources for future hybrid breeding. In contrast, the matK tree did not provide any phylogenetic information for hybrids but revealed a more reliable phylogenetic relationship conforming to the characteristics of traditional taxonomy. For convenience, a circular phylogenetic tree combining the main plant taxonomic features was constructed. As shown in the following Figure 1 and Supplementary Figure 1, *E. commutata* formed a distinct clade alone with a 98% bootstrap; *E. angustifolia* and *E. mollis* formed a clade with only 60% bootstrap; *E. umbellata*, *E. multiflora*, and *E. Montana* also formed a distinct clade with a 90% bootstrap; *E. bockii, E. henryi, E. pungens, E. conferta*, and *E. lanceolata* formed a distinct clade with a 95% bootstrap; and the remaining species of *E. glabra, E. macrophylla* and another haplotype of *E. pungens* formed a clade. Referring to the basic taxonomic features of plants such as deciduous, evergreen, flowering, and fruiting, a phylogenetic grouping model was constructed to divide the *Elaeagnus* species into four groups. Along the molecular phylogeny, the results showed that the maternal inheritance of *Elaeagnus* was characterized by the evolution of plant life forms from deciduous shrubs to erect or climbing evergreen plants. Thus, the characteristic DNA sequences reflected the dispersion of major morphological features.

When the phylogenetic tree was constructed, we also checked in time that some species were displayed to not be monophyletic. It was not surprising but instead regarded as a reasonable result, because the public DNA sequences come from different countries and laboratories, and there would inevitably be analytical interferences within acceptable limits due to sequencing, species identification, or other issues. According to the formulated principle at the beginning of sequence analysis, we retained all haplotypes of the same species for the next phylogenetic analysis. Thus, a small number of potentially outlier sequences (errors may arise from species identification errors or sequencing errors) must be presented on the phylogenetic tree. Therefore, critical thinking required us to first decide whether the phylogenetic tree is credible and usable and whether the results of genetic analysis can positively promote the next step of morphological grouping work. Practically, morphological grouping models were constructed mainly based on the clades supported by the branches of the phylogenetic tree with high confidence. A bootstrap value is the most reliable parameter to evaluate the phylogenetic tree and reliability of the branches. In general practice, branches with a bootstrap value greater than 95% will be explicitly discussed in the results, while branches with a bootstrap value less than 70% are considered low confidence and not discussed. We checked again if the species seem to not be monophyletic. For the matK sequences, we only found that only one haplotype (*E. umbellata*, MN599114) formed a non-monophyletic with a bootstrap value above 95%; For ITS, all non-monophyletic branch bootstrap values that can be found in the revised high-resolution Supplementary Figure 1 are less than 70%. In conclusion, the clades supported by the phylogenetic tree are invaluable for further analysis of genetic classification.

### Orthogonal Projections to Latent Structures Discriminant Analysis Model Established for Clustering China *Elaeagnus* L.

**FIGURE 2:**
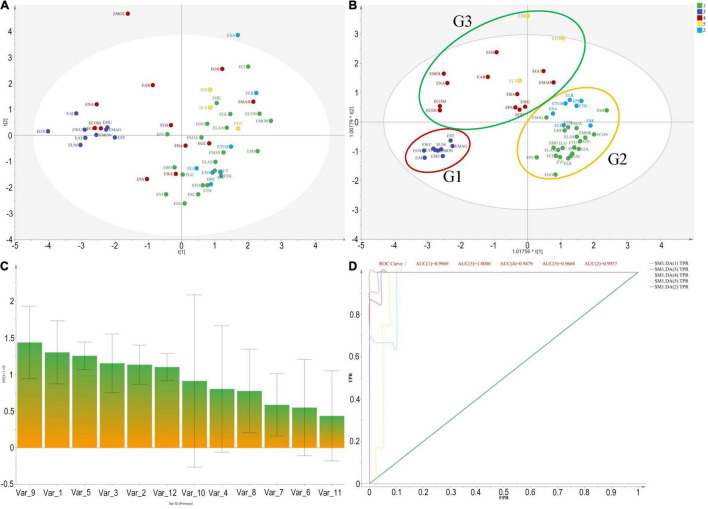
Cluster analysis based on morphological characteristics and molecular phylogenetic tree for grouping 56 *Elaeagnus* species in China. **(A)** Pure principal component analysis performed by using morphological traits, associated with sexual plant reproduction, were extracted and quantified according to the records of the Flora of China; **(B)** With the orthogonal partial least squares discriminant analysis (OPLS-DA) based on the phylogenetic tree of matK sequences, 56 species were well clustered into 3 groups, which were marked with G1, G2, and G3; **(C)** The variable importance for the projection (VIP) summarizing the importance of the variables, and this plot is sorted to display larger VIPs to the left. The top 5 contributing quantitative traits affecting the current clustering model are V9 (flower color), V1 (flowering), V5 (evergreen or deciduous), V3 (leaf size), and V2 (fruiting), respectively, (VIP > 1); and **(D)** The receiver operating characteristic (ROC) curve was used to evaluate the effectiveness of the grouping boundary.

The molecular phylogeny of the 14 *Elaeagnus* species presented a clear evolutionary relationship consistent with the plant taxonomy, and the divided clades could provide us with a basic grouping principle and an optimizing and training dataset for constructing a grouping model based solely on plant morphological data. The realization of this grouping model could undoubtedly provide us with an extremely practical breeding guide to deal with the dilemma that it is difficult to obtain more DNA sequences of larger-scale species in a short term. It is also believed that this dilemma cannot be easily solved with only sufficient financial support, because the field collection and species identification of more than 50 wild species cannot be completed in a short period of time. The preciousness of the breeder’s time is especially recognized in the perennial fruit tree breeding community. Therefore, we are looking forward to an invention and application of a morphological rapid grouping model based on molecular phylogenetic theory. In this study, a total of 12 morphological traits of 52 species, associated with sexual plant reproduction, were extracted and quantified according to the records of the FOC. Specimen information was used for a few species distributed abroad, such as *E. commutata* distributed in the United States and *E. montana* distributed in Japan. The 12 morphological traits were, respectively, related to the type of tree, leaf, the number, and color of the flowers, calyx shape, style character, and ecological type, among others. As shown in Figure 2A, the PCA method was first used, and the result was frustrating that the 56 species could not be clustered well for morphological grouping. However, the OPLS-DA model based on phylogenetic clades presented a clear clustering result. The result showed that 56 species were clustered well into three groups, which were marked with G1, G2, and G3 shown in Figure 2B. The results also pointed out the top 5 contributing morphological traits affecting the current clustering model, as shown in Figure 2C, which are V9 (flower color), V1 (flowering), V5 (evergreen or deciduous), V3 (leaf size), and V2 (fruiting), respectively. Among them, the flower color and flowering period played a decisive role in the morphological clustering of Chinese *Elaeagnus*. Furthermore, as shown in Figure 2D, the ROC curve revealed that the area under the ROC curve (AUC values) for both G1 and G2 both exceed 95%, indicating that the OPLS-DA model has a very well analysis effect for morphological grouping. Thus, the phylogenetic-based OPLS-DA model was proved to be an effective method for clustering the wild *Elaeagnus* plants as well as mining the key morphological features related to plant taxonomy.

### Difference Analysis of Morphological Traits Between Different Groups

**FIGURE 3:**
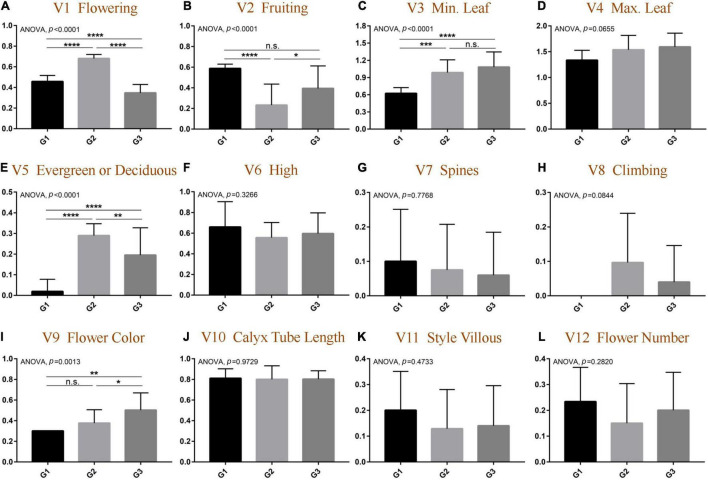
Comparative analysis of morphological differentiation at quantitative traits among different groups of genus *Elaeagnus*. **(A)** Flowering (V1); **(B)** Fruiting (V2); **(C)** Minimum leaf size (V3); **(D)** Maximum leaf size (V4); **(E)** Evergreen or deciduous (V5); **(F)** Plant height (V6); **(G)** Spines or absent (V7); **(H)** Climbing or not (V8); **(I)** Flower color (V9); **(J)** The length of calyx tube (V10); **(K)** Style villous (V11); and **(L)** the flower number (V12). (*N* > 9; *t*-test, ****p < 0.0001; ***p < 0.001; **p < 0.01; and *p < 0.05).

The data of morphological traits were normalized, and the results were consistent with the above OPLS-DA results. As shown in Figure 3 and Supplementary Figure 3, among the 12 morphological traits, five traits were significantly different among the three groups. First of all, the flower colors of the three groups were significantly different (ANOVA, *p* = 0.0013) and gradually changed from yellow to white from G1 to G3; the species divided into G2 had a relatively independent flowering and fruiting, and the flowering is concentrated in the fourth quarter (*t*-test, *p* < 0.0001), and the fruiting is in the first quarter of the next year (*t*-test, *p* < 0.05). Flowering uniformity was indeed a prerequisite for natural interspecific gene recombination, which directly leads to the rich species diversity of the G2 group. The results also implicated negatively that the types of calyx tube and style villous did not significantly contribute to group formation (ANOVA, *p* = 0.9729).

In summary, the most significant trait dispersion among different groups is flowering, while the G2 group of plants has better wild conditions for natural hybridization in China, mainly including the consistency of pollinating cycles and the cross-distribution of multiple species. Nonetheless, the geographic dispersion of different groups of *Elaeagnus* plants in China remains unclear. Further analysis of geographical dispersion is believed to be helpful in assessing cross-compatibility and uncovering potential geographic barriers.

### The Geographical Dispersion of China *Elaeagnus* Plants

**FIGURE 4:**
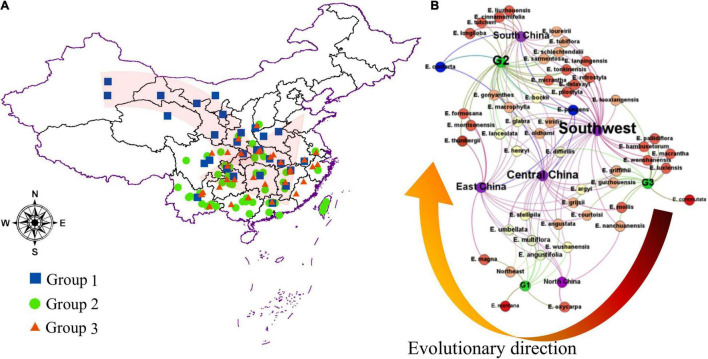
The geographical dispersion of the genus *Elaeagnus* in China. **(A)** Distribution of the main Chinese genus *Elaeagnus*, and three groups were divided by using the OPLS-DA model; **(B)** Neural Network analysis for distribution of different groups of genus *Elaeagnus* in China.

This study illustrated that there is no obvious geographic isolation among the three groups, except for a few species that were distributed on the plateau or crossed the Taiwan Strait. As shown in Supplementary Table 3 and Figure 4, the three geographic nodes include South China, Central China, and East China, which divided all species into upper and lower parts. Group G2 contained 28 species in the upper left corner of Figure 4B, and the groups G1 and G3 contained 24 species in the bottom right of Figure 4B. Therefore, the result is also consistent with some reported articles that the *Elaeagnus* species is native to Eurasia, northeastern Australia, and North America and distributes into the two river basins and the central plains of China along the Hexi corridor, and then to the southeast coast, southwest and north China (Qin et al., 2007; Du et al., 2020). The tree species dominated by deciduous shrubs formed the G3 group with narrower leaves, which were mainly distributed in high altitude or high latitude regions, including Gansu, Yunnan, and Guizhou provinces. The G1 group was also formed by some deciduous shrubs, but it was mainly distributed at low altitude regions (Figure 4A), and its leaf shape was significantly larger than that of the G3 group (Figure 3). The G2 group had reached the summit of molecular phylogeny and was distributed widely in the south of the Yellow River. Evergreen or semievergreen plants formed the G2 group. It was gratifying that the G2 group seems to have gathered all the Chinese underutilized fruits in *Elaeagnus* L., such as *E. pungens, E. glabra, E. macrophylla, Elaeagnus oldhami*, and *E. conferta.* In addition, the G2 group also figured out that a total of 10 wild species widely distributed in China have the potential to develop fruit crops. As shown in Table 2, there are three evergreen climbing shrubs, including *E. glabra*, *E. gonyanthes*, and *E. henryi*; two evergreen slightly climbing shrubs, including *E. macrophylla* and *E. conferta*; and five erect shrubs, including *E. lanceolata, E. delavayi, E. oldhami, E. pungens*, and *E. bockii.* Here, we have also sorted out the excellent dominant traits to prepare for the next collection of these wild resources. First, *E. conferta* could provide a founder species with a large fruit size, while *E. gonyanthes* could provide an important genetic resource with a long pedicel. *E. lanceolata* and *E. delavayi* could be used to domesticate hybrids without spines, and the five climbing shrubs could be used to develop high-yield crown type commercial cultivars that facilitate automated field management.

### Phylogenic Divergence Time Estimates

**FIGURE 5:**
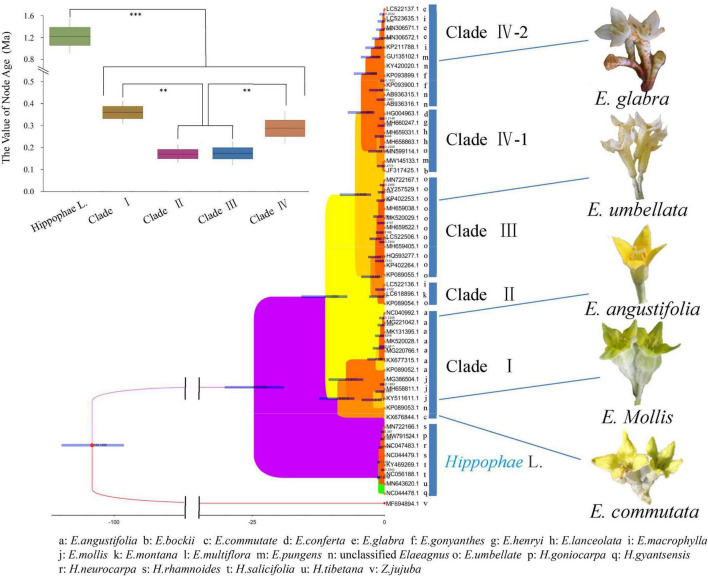
Time-calibrated Bayesian tree inferred from the matK sequences of the family Elaeagaceae and one outgroup species of Acer using BEAST. *t*-test, ***p < 0.001 and **p < 0.01.

Genetic diversity estimates were calculated by using the matK sequence. Then, we used the uncorrelated log-normal relaxed-clock model and the GTR + G model of DNA sequence evolution for each partition to accommodate mean branch rate variation among gene trees. As shown in Figure 5, a time-calibrated phylogeny was reconstructed by BEAST. *E. commutata* (KX676844) is native to Minnesota, North America and is an independent species as it formed a separate group with a bootstrap value of 98%, without combining with other groups. *E. angustifolia* and *E. mollis* formed a separate group and represented the deciduous clade together with *E. commutata* (Clade I). *E. umbellata* formed a separate deciduous group (Clade II). *Elaeagnus montana* and *E. multiflora* formed the last deciduous clade (Clade III). The remaining species formed a richly divided evergreen group. Among them, *E. lanceolata, E. conferta, E. pungens, E. henryi*, and *E. bockii* formed an evergreen clade (Clade IV-Part 1). *E. gonyanthes, E. macrophylla*, and *E. glabra* formed another evergreen clade (Clade IV-Part 2). The phylogeny of extant and fossil species showed support for our divergence time evaluation (Bell et al., 2010). The molecular clock inferred *Elaeagnus* originated between the early Oligocene and the middle Miocene at 11.22 Ma (95% HPD: 17.1–7.3 Ma).

## Discussion

### Cross-Latitude Geographical Dispersion Is Conducive to Developing Fruits of *Elaeagnus* L.

Fruits of *Hippophae* L. (Sea buckthorn) in the family Elaeagnaceae have been domesticated and cultivated in orchards, especially in China, Europe, Canada, and the United States with a long development history (Zhao, 1997; Rongsen et al., 2013). Fruits of *Elaeagnus* L. have similar characteristics to that of sea buckthorn (Li and Schroeder, 1996). Why did we spend so much energy on the cultivation and domestication of the future fruit crops of the genus *Elaeagnus* L.? First, sea buckthorn occurs as a native plant distributed only throughout the arid area of the northwestern part of the northern temperate zone, which directly restricted the large-scale cultivation as a fruit crop (Li et al., 2005); second, sea buckthorn is considered to be drought resistant (Zhang et al., 2022) is, therefore, considered an ideal plant that has been used for fighting soil erosion and also used in land reclamation (Ruan et al., 2013; Li G. et al., 2015), so the fruit used is just to supplement a by-product in many countries. Additionally, one of the major factors restricting the development of sea buckthorn is the berries type with difficult to transport and the limited shelf life of the fresh fruit. Whereas, the fruits of *Elaeagnus* L. have the potential to be popularized in a wide range of regions around the world due to their advantages in geographical dispersion and cross-latitude adaptability. The natural distribution of *Elaeagnus* L. species can extend from the northern temperate zone to subtropical and even tropical zones. This feature has practical implications for new approaches to fruit breeding in mid-low latitudes.

### New Grouping Model Is Expected to Guide the Construction of the Basis Pool

In general, traditional breeding methods, such as genetic selection or systematic breeding, are suitable for the initial domestication of wild plant resources (Dempewolf et al., 2017). Accurate characterization of the available genetic basis pool is therefore important in breeding programs and essential for the protection of future property right over new cultivars (Adebayo et al., 2009; Viruel et al., 2021). When the founder species has been determined, how to assess the taxonomic and phylogenetic position and finding the closely related species is very important to examine the cross-compatibility between them. In this study, Chinese *Elaeagnus* species were investigated first by using DNA marker regions of ITS and matK sequences. The traditional plant taxonomy was used to correct the results of molecular phylogenetic inferences. Phylogenetic relationships reconstructed with the matK sequences of the chloroplast genome suggested a clear characteristic of maternal inheritance. The present results may be interpreted as limited pieces of evidence, which supported that the phylogenetic analysis and the clustering of morphological traits may help to construct an effective population pool and revealed the genetic basis of a set of elite parents for breeding practice. The construction of genetic pools mainly relies on the breeder’s clear ideas and the grasp of breeding objectives, as well as the genetic collection of wild relatives with excellent morphological traits (Mars, 2000). However, in the absence of sufficient DNA sequences, how do we analyze the dispersive pattern only by using the morphological traits? In this study, the novel clustering model innovative provided excellent insights for understanding the evolution and biogeography of *Elaeagnus* L. in China. First, the phylogenetic positions of successful hybrids provided reference examples to gain the genetic characteristics and distribution patterns of hybrid compatible species and second, statistics and OPLS-DA model clustering 12 quantitative traits indicated natural dispersion of different species clusters. In addition, the current inference directly excavated 10 potential wild species in line with the breeding aims for further improvement and domestication.

### Multilateral Cross-Species Interactions of Morphology and Ecology Within Low Latitude Distributed Tribes

Previous phylogenetic studies using several gene regions revealed three well-supported clades in the genus *Elaeagnus* L. (Liu et al., 2019; Alexandrov and Karlov, 2021). Similarly, our results indicated that both MP and ML trees showed three or four well-supported clades in the genus *Elaeagnus* L. For the first time, we used the matK sequence of the DNA marker region of the chloroplast genome, and the obtained molecular phylogeny was concordant with the morphological taxonomy. As shown in Figure 5, the molecular clock inferred *Elaeagnus* originated between the early Oligocene and the middle Miocene at 11.22 Ma. In a macrofossil record, a single living and fossil species of *Elaeagnus*, namely *E. commutata*, is native to western and boreal North America, suggesting that the lineage had Asian ancestry and entered North America *via* the Bering land bridge (Su et al., 2014). As the first confirmed leaf fossil record in *Elaeagnus*, *Elaeagnus tibetensis* demonstrated that this genus was already distributed in the Qinghai-Tibet Plateau by the late Miocene (about 10∼5 Ma; Wang et al., 2006; Su et al., 2014). The diversity of *Elaeagnus* in this region may be closely associated with the uplift of the Qinghai-Tibet Plateau. This is consistent with our estimate that the evergreen group began to separate from deciduous shrubs at 5.58 Ma years ago. The Chinese current evergreen *Elaeagnus* species originated at 0.3 Ma. The uplift of the Qinghai-Tibet Plateau gradually strengthened the monsoonal climate in eastern Asia during the Neogene (Zhisheng et al., 2001; Liu and Yin, 2002). Because of the strongly seasonal precipitation, *Elaeagnus* tend to have a high density of scales on both sides of young leaves, but the adaxial scales tend to detach when the leaves are fully expanded. The hydrophobic of leaf properties may be an important condition for the migration of *Elaeagnus* from high latitudes to low latitudes, but how the interspecies evolution after living in low latitudes has not been reported now. Interestingly, intraspecific diversity was observed in this study. *E. pungens, E. macrophylla*, and *E. glabra* have abundant haplotypes and are clustered within different phylogenetic branches (Supplementary Figure 1) in Clade IV. Additionally, as shown in Figure 5, the perianth color of *Elaeagnus* evolved from dark yellow or green to white, and the eight species we investigated in Clade IV all represent flowers of the same or similar white color (Supplementary Figure 2). Thus, the clade IV is a large evergreen type woody shrub, and the white flowers are hermaphrodite and are pollinated by honey bees. Hypothetically, pollinator diversity (Ollerton, 2017) at low latitudes may facilitate the exchange of genetic information across *Elaeagnus* species. In conclusion, the phylogenetic analysis reconstructed the preliminary pieces of evidence for the geographic dispersion and cross-species interactions at low-latitude of *Elaeagnus* species in China.

## Conclusion

The widely distributed wild germplasm resources of *Elaeagnus* L. in China are the core conditions for developing new berry crops of fruit trees. Aiming at how to efficiently construct competent genetic basis pools, in this study, we constructed a phylogenetic tree using the DNA marker regions in the chloroplast genome and a novel OPLS-DA model for morphological clustering according to the phylogenetic tribes. In this study, the Chinese *Elaeagnus* species were divided into three groups, and we have pioneered the phylogenetic position of the hybrid species, which can have far-reaching significance for the construction of the basic population pools. The results summarized that a total of 10 wild species widely distributed in China have the potential to develop fruit crops. Furthermore, the results here are the first time to visualize the natural co-evolution of *Elaeagnus* species in low-latitude of China from a new perspective through phylogenetic analysis and geographical dispersion. We also analyzed the phylogenetic status of the parental materials of the reported interspecific hybridization and its distribution in China. In conclusion, we have provided a list of species that are solemnly recommended for systematic selection or cross-breeding to create new fruit-type *Elaeagnus* crops.

## Data Availability Statement

The original contributions presented in the study are included in the article/Supplementary Material, further inquiries can be directed to the corresponding authors.

## Author Contributions

CC, ZW, and HH: conceptualization. CC, CW, LY, and SF: methodology. CC, CW, and SF: software. LY and SF: validation. SF: formal analysis and visualization. CC: investigation, data curation, writing—original draft preparation, and project administration. SF and CC: resources. CC and HH: writing—review and editing. HH: supervision and funding acquisition. All authors have read and agreed to the published version of the manuscript.

## Conflict of Interest

The authors declare that the research was conducted in the absence of any commercial or financial relationships that could be construed as a potential conflict of interest.

## Publisher’s Note

All claims expressed in this article are solely those of the authors and do not necessarily represent those of their affiliated organizations, or those of the publisher, the editors and the reviewers. Any product that may be evaluated in this article, or claim that may be made by its manufacturer, is not guaranteed or endorsed by the publisher.

## Footnotes

1
https://www.cvh.ac.cn
