# Supplementary material for: Phylogenetic Analysis of Elaeagnus L. in China: A Basis for Genetic Improvement of a Berry Crop
Source: Front Plant Sci. 2022 Jun 9;13:899079. doi: 10.3389/fpls.2022.899079 (PMC9223766; doi:10.3389/fpls.2022.899079)
Supplement: Supplementary file 1 [file Data_Sheet_1.docx]

Supplementary Figures


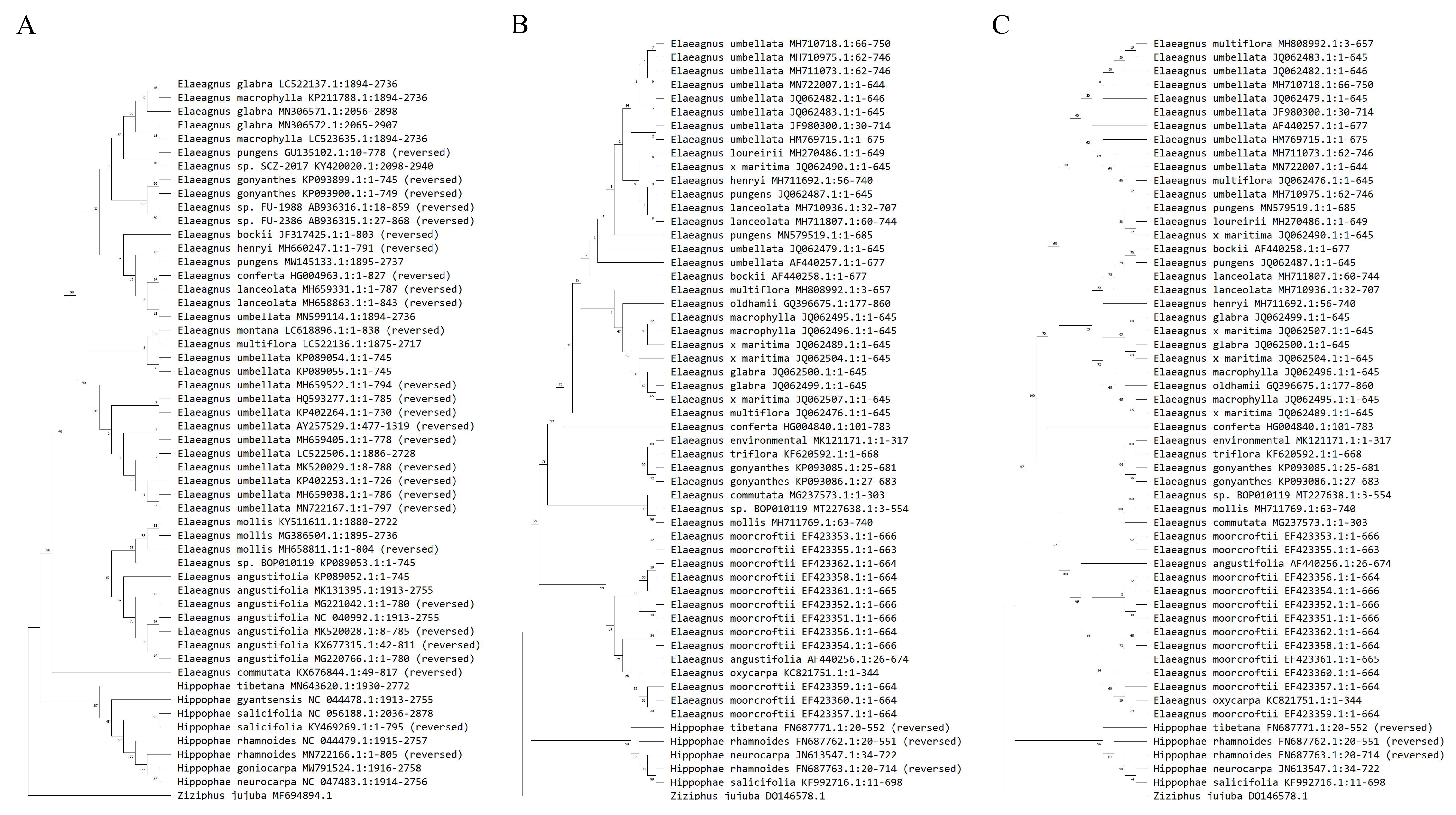


Supplementary Figure 1: the strict consensus trees constructed by using the maximum parsimony (MP) based on the ITS sequences and the maximum likelihood (ML) based on the ITS and matK sequence, with *Ziziphus jujuba* as the outgroup. A: polygenetic tree using matK sequences by ML method; B: polygenetic tree using ITS sequences by ML method; C: polygenetic tree using ITS sequences by MP method.


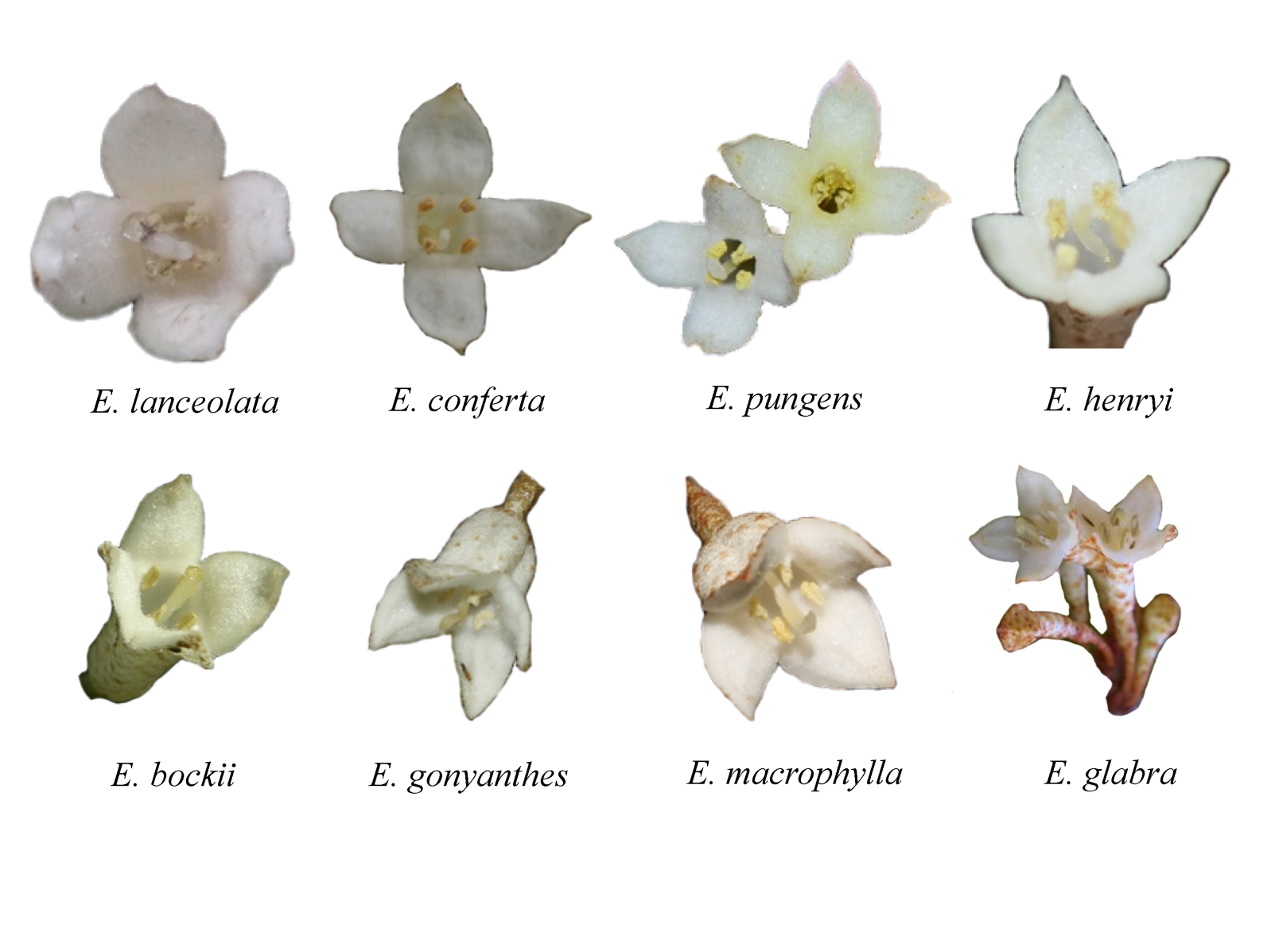


Supplementary Figure 2: the 8 *Elaeagnus* species we investigated in Clade IV, all represent flowers of the same or similar white color.


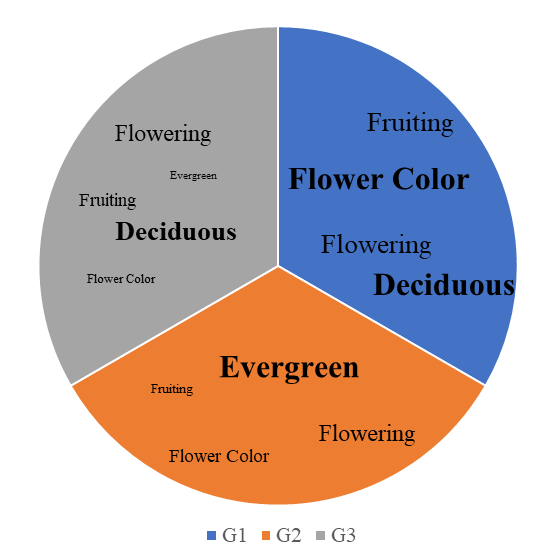


Supplementary Figure 3: similarity presentation of morphological features between species in different groups (The largest font indicates that the morphological feature is the same or most similar within the different species in the group.)

Supplementary Tables

**Supplementary Table 1: detailed information of sampling sequences used for phylogenetic study**

| GenBank | Sequence Length (bp) | Species | Address | Sequencing Technology | JOURNAL |
| --- | --- | --- | --- | --- | --- |
| MF694894.1 | 811 | *Z.jujuba* | Department of Biochemistry, University of Agriculture Faisalabad, 21 DC Road, Gujranwala, Punjab 52250, Pakistan | - | Unpublished |
| NC047483.1 | 156316 | *H.neurocarpa* | College of Eco-Environmental Engineering, Qinghai University, NingDa Road 251, Xining, Qinghai 810016, China | Illumina | Mitochondrial DNA B Resour 4 (1), 2048-2049 (2019) |
| MW791524.1 | 156312 | *H.goniocarpa* | School of Ecology and Environmental Sciences, Yunnan University, East Outer Ring Road, Kunming, Yunnan 650500, China | - | Unpublished |
| MN722166.1 | 805 | *H.rhamnoides* | Northwest University, Taibai north Rd.229, Xi'an City, Shaanxi Province, China., Xi'an, Shaanxi 710069, China | Sanger dideoxy sequencing | Unpublished |
| NC044479.1 | 156415 | *H.rhamnoides* | Research Institute of Forestry, Chinese Academy of Forestry, Xiangshan Street, Beijing, Beijing 100091, China | Illumina | J For Res (Harbin) (2019) In press |
| KY469269.1 | 795 | *H.salicifolia* | Department of Botany, University of Delhi, Delhi 110007, India | Sanger dideoxy sequencing | Unpublished |
| NC056188.1 | 155420 | *H.salicifolia* | School of Life Sciences Fudan University, Ministry of Education Key Laboratory of Biodiversity Science and Ecological Engineering, 2005 Songhu Rd., Shanghai, Shanghai 200438, P. R. China | Illumina | Unpublished |
| NC044478.1 | 155260 | *H.gyantsensis* | Research Institute of Forestry, Chinese Academy of Forestry, Xiangshan road, Beijing, Beijing 100091, China | Illumina | J For Res (Harbin) (2019) In press |
| MN643620.1 | 155810 | *H.tibetana* | College of Eco-Environmental Engineering, Qinghai University, NingDa Road 251, Xining, Qinghai 810016, China | Illumina | Unpublished |
| KX676844.1 | 817 | *E.commutata* | Botany, University of British Columbia, 3529-6270 University Blvd., Vancouver, BC V6T 1Z4, Canada | - | Unpublished |
| MG220766.1 | 780 | *E.angustifolia* | Canadian Center for DNA Barcoding, Center for Biodiversity Genomics, 50 Stone Rd E, Guelph, Ontario N1G2W1, Canada | - | Appl Plant Sci 5 (12) (2017) |
| KX677315.1 | 811 | *E.angustifolia* | Botany, University of British Columbia, 3529-6270 University Blvd., Vancouver, BC V6T 1Z4, Canada | - | Unpublished |
| MK520028.1 | 785 | *E.angustifolia* | Botany, University of Wisconsin-Madison, 430 Lincoln Dr, Madison, WI 53706, USA | - | Unpublished |
| NC040992.1 | 150546 | *E.angustifolia* | Research Institute of Forestry, Chinese Academy of Forestry, NO. 1 Dongxiaofu, Xiangshan RD, Haidian district, Beijing, Beijing 100091, China | - | Unpublished |
| MG221042.1 | 780 | *E.angustifolia* | Canadian Center for DNA Barcoding, Center for Biodiversity Genomics, 50 Stone Rd E, Guelph, Ontario N1G2W1, Canada | - | Appl Plant Sci 5 (12) (2017) |
| MK131395.1 | 150546 | *E.angustifolia* | Research Institute of Forestry, Chinese Academy of Forestry, NO. 1 Dongxiaofu, Xiangshan RD, Haidian district, Beijing, Beijing 100091, China | - | Unpublished |
| KP089052.1 | 745 | *E.angustifolia* | State Key Laboratory of Systematic and Evolutionary Botany, Institute of Botany, Chinese Academy of Sciences, No. 20 Nanxincun, Xiangshan, Beijing, Beijing, Beijing 100093, China | - | Sci Rep 5, 8348 (2015) |
| KP089053.1 | 745 | *Elaeagnus sp.* BOP010119 | State Key Laboratory of Systematic and Evolutionary Botany, Institute of Botany, Chinese Academy of Sciences, No. 20 Nanxincun, Xiangshan, Beijing, Beijing, Beijing 100093, China | - | Sci Rep 5, 8348 (2015) |
| MH658811.1 | 804 | *E.mollis* | Key Laboratory of Plant Resources Conservation and Sustainable Utilization, South China Botanical Garden, The Chinese Academy of Sciences, Xingke Road 723, Guangzhou, Guangdong 510650, China | - | Unpublished |
| MG386504.1 | 151354 | *E.mollis* | College of Life Sciencs, Northwest University, North Taibai Road 229, Xi'an, Shaanxi 710069, China | - | Unpublished |
| KY511611.1 | 151428 | *E.mollis* | College of Forestry, Northwest A&F University, Taicheng Road #3, Yangling, Shaanxi Province 712100, China | - | Unpublished |
| MN722167.1 | 797 | *E.umbellata* | Northwest University, Taibai north Rd.229, Xi'an City, Shaanxi Province, China., Xi'an, Shaanxi 710069, China | Sanger dideoxy sequencing | Unpublished |
| MH659038.1 | 786 | *E.umbellata* | Key Laboratory of Plant Resources Conservation and Sustainable Utilization, South China Botanical Garden, The Chinese Academy of Sciences, Xingke Road 723, Guangzhou, Guangdong 510650, China | - | Unpublished |
| KP402253.1 | 726 | *E.umbellata* | Botany, Smithsonian Institution, MRC 166 PO Box 37012, Washington, DC 20013-7012, USA | - | Unpublished |
| MK520029.1 | 788 | *E.umbellata* | Botany, University of Wisconsin-Madison, 430 Lincoln Dr, Madison, WI 53706, USA | - | Unpublished |
| LC522506.1 | 152261 | *E.umbellata* | Yonguk Kim Jeollanamdo Institute of Natural Resources Research, Natural Resources Research; 288, Woodland-gil, Anyang-myeon, Jangheung-gun, Jeollanam-do 59338, Korea | Illumina HiSeq | Unpublished |
| MH659405.1 | 778 | *E.umbellata* | Key Laboratory of Plant Resources Conservation and Sustainable Utilization, South China Botanical Garden, The Chinese Academy of Sciences, Xingke Road 723, Guangzhou, Guangdong 510650, China | Sanger dideoxy sequencing | Unpublished |
| AY257529.1 | 1518 | *E.umbellata* | Biological Sciences, University of Maine, 261 Hitchner Hall, Orono, ME 04469-5751, USA | - | Am. J. Bot. 90 (12), 1758-1776 (2003) |
| KP402264.1 | 730 | *E.umbellata* | Botany, Smithsonian Institution, MRC 166 PO Box 37012, Washington, DC 20013-7012, USA | - | Unpublished |
| HQ593277.1 | 785 | *E.umbellata* | Biology, Columbus State University, 4225 University Avenue, Columbus, GA 31907, USA | - | Unpublished |
| MH659522.1 | 795 | *E.umbellata* | Key Laboratory of Plant Resources Conservation and Sustainable Utilization, South China Botanical Garden, The Chinese Academy of Sciences, Xingke Road 723, Guangzhou, Guangdong 510650, China | Sanger dideoxy sequencing | Unpublished |
| KP089055.1 | 745 | *E.umbellata* | State Key Laboratory of Systematic and Evolutionary Botany, Institute of Botany, Chinese Academy of Sciences, No. 20 Nanxincun, Xiangshan, Beijing, Beijing, Beijing 100093, China | - | Sci Rep 5, 8348 (2015) |
| KP089054.1 | 745 | *E.umbellata* | State Key Laboratory of Systematic and Evolutionary Botany, Institute of Botany, Chinese Academy of Sciences, No. 20 Nanxincun, Xiangshan, Beijing, Beijing, Beijing 100093, China | - | Sci Rep 5, 8348 (2015) |
| LC522136.1 | 152267 | *E.multiflora* | Yonguk Kim Jeollanamdo Institute of Natural Resources Research, Natural Resources Research; 288, Woodland-gil, Anyang-myeon, Jangheung-gun, Jeollanam-do 59338, Korea | - | Unpublished |
| LC618896.1 | 840 | *E.montana* | Manabu Ogiso Aichi Gakuin University, Division of Liberal Arts and Sciences; 12 Araike Iwasaki-cho, Nisshin, Aichi 470-0195, Japan | - | Unpublished |
| MN599114.1 | 152206 | *E.umbellata* | Life Science College, Neijiang Normal University, Dongtong Road 705, Neijiang, Sichuan 641112, China | - | Unpublished |
| MH658863.1 | 843 | *E.lanceolata* | Key Laboratory of Plant Resources Conservation and Sustainable Utilization, South China Botanical Garden, The Chinese Academy of Sciences, Xingke Road 723, Guangzhou, Guangdong 510650, China | - | Unpublished |
| MH659331.1 | 787 | *E.lanceolata* | Key Laboratory of Plant Resources Conservation and Sustainable Utilization, South China Botanical Garden, The Chinese Academy of Sciences, Xingke Road 723, Guangzhou, Guangdong 510650, China | Sanger dideoxy sequencing | Unpublished |
| HG004963.1 | 834 | *E.conferta* | Plant Geography Lab, Xishuangbanna Tropical Botanical Garden, Menglun, 666303, CHINA | - | Unpublished |
| MW145133.1 | 152218 | *E.pungens* | Department of Gastroenterology, Affiliated Hangzhou First People's Hospital, Zhejiang University School of Medicine, No. 261 Huansha Street, Hangzhou, Zhejiang 310006, China | Illumina | Unpublished |
| MH660247.1 | 791 | *E.henryi* | Key Laboratory of Plant Resources Conservation and Sustainable Utilization, South China Botanical Garden, The Chinese Academy of Sciences, Xingke Road 723, Guangzhou, Guangdong 510650, China | Sanger dideoxy sequencing | Unpublished |
| JF317425.1 | 999 | *E.bockii* | Key Laboratory of Biodiversity and Biogeography, Plant Germplasm and Genomics Center, Germplasm Bank Sciences, 132, Lanhei Road, Kunming, Yunnan 650204, China | - | Mol. Phylogenet. Evol. 60 (1), 21-28 (2011) |
| AB936315.1 | 981 | *Elaeagnus sp.* FU-2386 | Hironori Toyama Kyushu University, Biology; 6-10-1, Hakozaki, Fukuoka, Fukuoka 812-8581, Japan | - | Unpublished |
| AB936316.1 | 930 | *Elaeagnus sp.* FU-1988 | Hironori Toyama Kyushu University, Biology; 6-10-1, Hakozaki, Fukuoka, Fukuoka 812-8581, Japan | - | Unpublished |
| KP093900.1 | 749 | *E.gonyanthes* | South China Botanical Garden, Key Laboratory of Plant Resources Conservation and Sustainable Utilization, Xingke Road 723, Guangzhou, Guangdong 510650, China | Sanger dideoxy sequencing | Divers. Distrib. 21 (2), 188-199 (2015) |
| KP093899.1 | 745 | *E.gonyanthes* | South China Botanical Garden, Key Laboratory of Plant Resources Conservation and Sustainable Utilization, Xingke Road 723, Guangzhou, Guangdong 510650, China | Sanger dideoxy sequencing | Divers. Distrib. 21 (2), 188-199 (2015) |
| KY420020.1 | 126326 | *Elaeagnus sp.* SCZ-2017 | Plant Germplasm and Genomics Center, Germplasm Bank of Wild Species, Kunming Institute of Botany, 132, Lanhei Road, Kunming, Yunnan 650201, China | - | New Phytol. 214 (3), 1355-1367 (2017) |
| GU135102.1 | 778 | *E.pungens* | Florida Museum of Natural History, University of Florida, P.O. Box 117800, Gainesville, FL 32611-7800, USA | - | Unpublished |
| LC523635.1 | 152224 | *E.macrophylla* | Yonguk Kim Jeollanamdo Institute of Natural Resources Research, Natural Resources Research; 288, Woodland-gil, Anyang-myeon, Jangheung-gun, Jeollanam-do 59338, Korea | Illumina HiSeq | Unpublished |
| MN306572.1 | 152529 | *E.glabra* | The Experimental Station of the Research Institute of Tropical Forestry, Chinese Academy of Forestry, The Research Institute of Tropical Forestry, Chinese Academy of Forestry, Jianfeng Town, Ledong Li Autonomous County, Hainan 572500, China | - | Mitochondrial DNA B Resour 5 (1), 288-289 (2020) |
| MN306571.1 | 152555 | *E.glabra* | The Experimental Station of the Research Institute of Tropical Forestry, Chinese Academy of Forestry, The Research Institute of Tropical Forestry, Chinese Academy of Forestry, Jianfeng Town, Ledong Li Autonomous County, Hainan 572500, China | - | Mitochondrial DNA B Resour 5 (1), 288-289 (2020) |
| KP211788.1 | 152224 | *E.macrophylla* | College of Life Science, Yeungnam University, Dae-Dong, Gyeongsan-Si, Gyeongsangbuk-Do 712-749, Korea | Illumina | PLoS ONE 10 (9), E0138727 (2015) |
| LC522137.1 | 152227 | *E.glabra* | Yonguk Kim Jeollanamdo Institute of Natural Resources Research, Natural Resources Research; 288, Woodland-gil, Anyang-myeon, Jangheung-gun, Jeollanam-do 59338, Korea | Illumina HiSeq | Unpublished |

**Supplementary Table 2: the reference sequences of ITS and matK used in this study**

| Barcode | Position | Sequence information |
| --- | --- | --- |
| matK | 1  80  159  238  317  396  475  554  633 | TCCACATATATGTGCAAATCGATCGATAATATCCAAATCTGACGAATCGGCCCAGATTGACTTACTAACCGGATGTCCTAATCCATTACAAAATTTCATTTTAGCTAACGATCCAATTAGAGGACTAATTGGAACTAATGTATCAAGTCTCTTCATAGCATTATCTATTAGAAATGCATTTTTTAGCATTTGACTCCGTACCACCGAAAGGTTTAGTCGTATACTTGAAAAATAGCCCAAAAAAATGAGGGAATGCTTGGATAATTGGTTTATATAGATCCTTGCTGGTTGAGACCACACATAAAAATAAGATTGCCATAAATGGATAAGGTAATATTTCCATTTATTCATCAGAAGAGGTGTATCTTTTGAAGCCAAAATAGATTTTCCTCGATATCGAACATAATGCATGAAAGGGGCCTTGAAGAACCACGGAGTAACTGAAAAATCATTAACAATGACTTCTTCTACAGGATGTTTGATTTTTGCATAGAAAAAAATTCGTTCAAAAAAAGATCCAAAAGACGTTAATCGTAAATGAGAAGGTTTGTTACGAAGAAAAAGTAAGATAGATTCGTATTCACAAACATGAGAATTATATAGGAACAATAAAAATCTGGATTACTTTTTGAAAAAATAAAAAAAGAACTCTTTGGGATAATAATACTATTCCAATACTTGTGAAGATA |
| ITS | 1  80  159  238  317  396  475 | GGCTTGGATGGCCTCATTGTTGGTTGGAATGTTGTTTGATGCTGTCCCTTGCTCATGCATGTGGTAGGCATCGCAACATGCCCTAAAAACTAACCCCGGCGCAAATTGCGCCAAGGAACTCTAACGAATGAGTCACGGTGCCCATATGAGAGTTGCGTCGTATTCGATATGTCAAAAATGACTCTCGGCAACGGATATCTCGGCTCTCGCATCGATGAAGAACGTAGCGAAATGCGATACTTGGTGTGAATTGCAGAATCCCGTGAACCATCGAGTCTTTGAACGCAAGTTGCGCCCGAAGCCATCCGGTTGAGGGCACGTCTGCCTGGGCGTCACACACCGTTGCCCTCCTAACACCTCGTGCCTTTAGGCTATGTCGGTTGTGAAGGCGTACATTGGCTTCCCATGGCTTTGTCTTGTGGTTGGCCCAAATTCTAGTCATTGGTGACCAGTGCCACGACAATGGTGGATGTCGAACCTTCGGTGCCCCGTCATGGGTTCTGGTCGTCCATGATGCAAGGACCCAATGCATCCAATTTGA |

**Supplementary Table 3: detailed information of species distribution for studying geographical dispersion**

| NO. | Group | Longitude(E/W) | Latitude (N) | Species |
| --- | --- | --- | --- | --- |
| 1 | G1 | 112 | 34 | *E.umbellata*(EUM) |
| 2 |  | 108.7 | 34.5 |  |
| 3 |  | 104.8 | 34.2 |  |
| 4 |  | 110.8 | 35.2 |  |
| 5 |  | 133.4 | 35.4 |  |
| 6 |  | 118 | 30.3 | *E.multiflora* (EMU) |
| 7 |  | 114.5 | 30 |  |
| 8 |  | 116 | 29 |  |
| 9 |  | 103.3 | 30 | *E.angustata*(EAT) |
| 10 |  | 102.3 | 25 |  |
| 11 |  | 111.4 | 32 |  |
| 12 |  | 89.1 | 42 | *E.angustifolia*(EAI) |
| 13 |  | 106 | 37.2 |  |
| 14 |  | 102.2 | 38.8 |  |
| 15 |  | 114.2 | 34.7 |  |
| 16 |  | 111.5 | 28.2 | *E.magna*(EMAG) |
| 17 |  | 112.9 | 30.7 |  |
| 18 |  | 115.3 | 26.7 |  |
| 19 |  | 113.3 | 24.2 |  |
| 20 |  | 89.06 | 40 | *E.oxycarpa*(EOX) |
| 21 |  | 97.9 | 37 |  |
| 22 |  | 96.9 | 40 |  |
| 23 |  | 104.9 | 40 |  |
| 24 |  | 104.1 | 30.5 | *E.stellipila*(EST) |
| 25 |  | 139.8 | 37.0 | *E.montana* (EMON) |
| 26 |  | 106.6 | 29.2 | *E.wushanensis*(EWU) |
| 27 |  | 107.6 | 28.1 |  |
| 28 |  | 111 | 32 |  |
| 29 |  | 109 | 31.4 |  |
| 30 |  | 108.12 | 33.14 |  |
| 31 | G2 | 112.8 | 24.2 | *E.glabra* (EGL) |
| 32 |  | 109.5 | 24.9 |  |
| 33 |  | 107.4 | 26.9 |  |
| 34 |  | 115.1 | 26.3 |  |
| 35 |  | 120.9 | 23.78 | *E.macrophylla* (EMAL) |
| 36 |  | 119.6 | 31.8 |  |
| 37 |  | 120 | 29 |  |
| 38 |  | 134.08 | 35.1 | *E.macrophylla* (EMAY) |
| 39 |  | 112.2 | 26.14 | *E.gonyanthes* (EGO) |
| 40 |  | 113.3 | 24.3 |  |
| 41 |  | 109.4 | 24.4 |  |
| 42 |  | 104.3 | 24 |  |
| 43 |  | 111.9 | 28.7 | *E.pungens* (EPU) |
| 44 |  | 112 | 31 |  |
| 45 |  | 115.3 | 28.7 |  |
| 46 |  | 103.7 | 31.1 | *E.bockii*(EBO) |
| 47 |  | 110.4 | 32.9 |  |
| 48 |  | 111.3 | 35.7 |  |
| 49 |  | 106.8 | 28.2 |  |
| 50 |  | 106.3 | 29.7 | *E.henryi* (EHE) |
| 51 |  | 107.5 | 27.5 |  |
| 52 |  | 110.9 | 28.6 |  |
| 53 |  | 110.7 | 30.8 |  |
| 54 |  | 102 | 25 | *E.conferta* (EON) |
| 55 |  | 107.9 | 23.5 |  |
| 56 |  | 114.9 | 27.2 |  |
| 57 |  | 110.7 | 30.9 |  |
| 58 |  | 108.3 | 34 | *E.lanceolata* (ELAA) |
| 59 |  | 101.2 | 25.2 |  |
| 60 |  | 105.7 | 31.7 |  |
| 61 |  | 111.6 | 31.7 |  |
| 62 |  | 109.9 | 22.6 | *E.cinnamomifolia* (ECI) |
| 63 |  | 107 | 29.5 | *E.delavayi* (EDL) |
| 64 |  | 105.9 | 27.15 |  |
| 65 |  | 102.4 | 25.2 |  |
| 66 |  | 120.7 | 23 | *E.formosana* (EFO) *E.thunbergii* (ETH) |
| 67 |  | 121.2 | 24.35 |  |
| 68 |  | 101 | 25 | *E.lanpingensis* (ELAS) |
| 69 |  | 107 | 27 | *E.longiloba* (ELG) *E.retrostyla* (ERE) |
| 70 |  | 102.9 | 24.08 | *E.micrantha* (EMI) |
| 71 |  | 120.8 | 23.7 | *E.morrisonensis* (EMOR) *E.oldhami* (EOL) |
| 72 |  | 114.3 | 22.4 | *E.tutcheri* (ETUT) |
| 73 |  | 112.8 | 22.8 |  |
| 74 |  | 99.4 | 26.5 | *E.viridis*(EVI) |
| 75 |  | 100.6 | 30.4 |  |
| 76 |  | 111.25 | 32 |  |
| 77 |  | 108.9 | 24.4 | *E.liuzhouensis* (ELI) *E.schlechtendalii* (ESC) |
| 78 |  | 102.3 | 25 | *E.loureirii* (ELR) |
| 79 |  | 110.4 | 24.3 |  |
| 80 |  | 113.7 | 24.2 |  |
| 81 |  | 105.3 | 28.4 | *E.pilostyla* (EPI) |
| 82 |  | 102.6 | 25.6 | *E.sarmentosa* (ESA) |
| 83 |  | 103.7 | 23.7 | *E.tonkinensis*(ETO) |
| 84 |  | 105 | 23.7 | *E.tubiflora* (ETUB) |
| 85 | G3 | 111 | 35.6 | *E.mollis* (EMOL) |
| 86 |  | 108.3 | 33.7 |  |
| 87 |  | 119.7 | 31.7 | *E.argyi* (EAR) |
| 88 |  | 119.2 | 29.6 |  |
| 89 |  | 115.9 | 29.17 |  |
| 90 |  | 111.5 | 32 |  |
| 91 |  | 114.5 | 30.2 |  |
| 92 |  | 104 | 23.8 | *E.bambusetorum* (EBA) |
| 93 |  | 118 | 30.6 | *E.courtoisi* (ECOU) |
| 94 |  | 119.9 | 29.9 |  |
| 95 |  | 115.9 | 28.8 |  |
| 96 |  | 113.4 | 31.3 |  |
| 97 |  | 102.9 | 25.5 | *E.griffithii* (EGH) |
| 98 |  | 116.8 | 25.6 |  |
| 99 |  | 117.9 | 27.4 | *E.grijsii* (EGS) |
| 100 |  | 107.7 | 27.7 | *E.guizhouensis* (EGU) |
| 101 |  | 110.5 | 28 |  |
| 102 |  | 102.8 | 25.6 | *E.macrantha* (EMAH) |
| 103 |  | 106.7 | 29.7 | *E.nanchuanensis* (ENA) |
| 104 |  | 104.4 | 31.6 | *E.wenshanensis* (EWE) |
| 105 |  | 106.6 | 27.7 |  |
| 106 |  | 103.1 | 25.8 | *E.pallidiflora* (EPA) |
| 107 |  | 105.5 | 26.6 | *E.difficilis* (EDI) |
| 108 |  | 109.8 | 25 |  |
| 109 |  | 106.7 | 31.7 |  |
| 110 |  | 108.7 | 31.5 |  |
| 111 |  | 111.2 | 31.8 |  |
| 112 |  | 113 | 31 |  |
| 113 |  | 115.2 | 27.3 |  |
| 114 |  | 109.7 | 25 |  |
| 115 |  | 113.2 | 24.4 |  |
| 116 |  | 112.2 | 27 |  |
| 117 |  | 107.2 | 24 | *E.luoxiangensis* (ELO) |
| 118 |  | 104 | 23.7 | *E.luxiensis* (ELX) |
| 119 |  | 114.2 (W) | 51.1 | *E.commutata* (ECOM) |
| 120 |  | 95.9 (W) | 47.4 |  |
| 121 |  | 145.8 (W) | 63.9 |  |
| 122 |  | 80.7 (W) | 51.8 |  |
| 123 |  | 12.9 | 55.5 |  |
| 124 |  | 15.9 | 59.1 |  |
| 125 |  | 27.6 | 57.8 |  |
| 126 |  | 83.1 | 54.9 |  |
